# Changes in Timing, Duration, and Symmetry of Molt of Hawaiian Forest Birds

**DOI:** 10.1371/journal.pone.0029834

**Published:** 2012-01-18

**Authors:** Leonard A. Freed, Rebecca L. Cann

**Affiliations:** 1 Department of Biology, University of Hawaii at Manoa, Honolulu, Hawaii, United States of America; 2 Department of Cell and Molecular Biology, University of Hawaii at Manoa, Honolulu, Hawaii, United States of America; University of Debrecen, Hungary

## Abstract

Food limitation greatly affects bird breeding performance, but the effect of nutritive stress on molt has barely been investigated outside of laboratory settings. Here we show changes in molting patterns for an entire native Hawaiian bird community at 1650–1900 m elevation on the Island of Hawaii between 1989–1999 and 2000–2006, associated with severe food limitation throughout the year beginning in 2000. Young birds and adults of all species took longer to complete their molt, including months never or rarely used during the 1989–1999 decade. These included the cold winter months and even the early months of the following breeding season. In addition, more adults of most species initiated their molt one to two months earlier, during the breeding season. Suspended molt, indicated by birds temporarily not molting primary flight feathers during the months of peak primary molt, increased in prevalence. Food limitation reached the point where individuals of all species had asymmetric molt, with different primary flight feathers molted on each wing. These multiple changes in molt, unprecedented in birds, had survival consequences. Adult birds captured during January to March, 2000–2004, had lower survival in four of five species with little effect of extended molt. Extended molt may be adaptive for a nutrient stressed bird to survive warm temperatures but not cool winter temperatures that may obliterate the energy savings. The changing molt of Hawaiian birds has many implications for conservation and for understanding life history aspects of molt of tropical birds.

## Introduction

Breeding and molting, in that order, are the primary stages of the annual cycle in most birds. Both activities are energetically expensive [1]–[6], and therefore usually separated in time [7]–[8]. The initiation of molt is fixed in time relative to the breeding season [9], so much so that the end of the breeding season is assumed to begin with molt for most species of birds [10]. There is also evidence of molt being timed as an endogenous cycle [10]–[11]. Thus any environmental change that alters the initiation or duration of molt reflects modifications of underlying physiology. With the intense interest in anthropocentric environmental changes on the annual cycle of birds, much evidence has been collected on mismatch between breeding seasons and phenology of food [12]–[14], and the advance of breeding seasons to match the new phenology [15]–[17]. Less attention has been directed on changes in molt [18], and the possible mismatch of molt with altered environmental conditions.

Any environmental disturbance that reduces food availability during the molting season can affect feather replacement, but the responses are different than those for breeding. Breeding problems from food limitation are associated with reduced reproductive success evidenced in lowered clutch size, fledging success, and condition of offspring [19]. Food limitation during the molting stage can be visually inferred and quantified by noting the presence of fault bars in wing and tail feathers in individual birds. Fault bars form from failure of the bird to deposit melanin in the growing feather for one or several days, and reflect nutritive stress when that feather was molted [20]–[21]. Major fault bars can lead to broken wing and tail feathers (illustrated in [22]), which in turn can generate problems for flight [23]. In addition, experimentally induced food limitation can lead to asymmetric molt, when different feathers on the two wings are being molted concurrently [24]–[25]. Normally, when birds molt primary flight feathers, the same feathers on each wing are replaced at the same time, a character maintained by natural selection [26]. Asymmetric molt can reduce maneuverability during flight [27]–[28].

Food limitation during molt can also change the timing or duration of the molting season. Previous studies of molt relative to food shortages suggest that birds can delay, suspend, or protract molt [29]–[33]. By replacing individual feathers more slowly (protracted molt), or suspending molt, an individual reduces daily energy expenditures, but extends the molting season. Also, with food limitation, birds can begin molting within the normal breeding season and continue throughout the normal molting season, a phenomenon termed early molt [34]–[36]. Wild birds could even start earlier and finish later if sufficiently food-limited. One study of molt in neotropical birds identified a year where birds initiated molt early in association with lower food [34], and an example from temperate birds showed that such molt improved survival [36]. Another study reported that experimentally induced protracted molt associated with malnutrition in temperate zone-adapted white-crowned sparrows, (*Zonotrichia leucophrys gambelii*), resulted in the normal duration of molt doubling in length [33].

Tropical birds generally have longer molting seasons than temperate birds. For example, the duration of molting of tropical passerine birds in Costa Rica ranged from 4 months to 10 months [37], much longer than the 58 days of the temperate species used in the malnutrition study [33]. Wild tropical birds, not constrained by migration or harsh winter weather, might have greater opportunity to replace feathers more slowly than temperate birds. However, there may also be costs of slowing the replacement process with protracted, extended, or suspended molt. These include molt-breeding overlap, which is usually avoided [38]. In addition, molting during months which had previously been avoided might interfere with the next breeding season.

Hawaiian forest birds at Hakalau Forest National Wildlife Refuge on the Island of Hawaii represent an exceptional opportunity to investigate the effect of an observed environmental change on the molting stage of the annual cycle of tropical species. We have presented evidence elsewhere that food limitation during the breeding season occurred and was due to exploitative competition with the introduced Japanese white-eye (*Zosterops japonicus*), which increased in density beginning in 2000 in a long-term study site [39]. Following the increase, all species of native birds measured had stunted growth of young with lower survival in most species [40], and the nestling overgrowth and seasonal variation in sex allocation adaptations of the endangered Hawaii akepa (*Loxops coccineus coccineus*) were dismantled [39], [41]–[42]. Severe food limitation was also indicated by unfulfilled frantic begging of females for food from their mate and reduced begging vocalizations in adults and fledglings [39].

Further disruption of the annual cycle of the native bird community carried over into the non-breeding months. Beginning in 2000, every species had increased prevalence of broken wing and tail feathers [22]. Between 2003–2005 every species had major fault bars in wing and tail feathers, defined as wide and extending to or almost to the shaft of the feathers, with prevalence ranging from 9 to 28% [22]. These fault bars were not caused by human handling because initially captured birds had them [22]. In addition, most native species had lower fat during the winter months [22]. Thus, native birds showed multiple lines of evidence of food limitation during the entire year. Here we document that the timing, duration, and symmetry of molt changed in parallel with the stunted growth of young birds previously analyzed.

## Methods

### Ethics statement

Mist-netting and bird handling were performed under a protocol approved by the University of Hawaii Institutional Animal Care and Use Committee (00-005-12). The research was approved by the relevant endangered species permits (FREELA-5 though 9, UHMNZA 10–11, TE799001-12 through 15), federal bird banding master permit 21864, state collecting permits (WL-89 through 06), and refuge special use permits (HAK-1-88, SUP-9-93, 56050, and 12516–99014, 030189, 99013, 00009, 01013).

### Study sites and species

Study sites at Hakalau Forest National Wildlife Refuge, Island of Hawaii, were located along an elevational gradient at 1900, 1770, and 1650 m in the southern section of the refuge where endangered species have their highest densities [43]. Community structure was very similar among sites [39]. The predominant canopy trees in the forest at all three sites were ohia-lehua (*Metrosideros polymorpha*) and koa (*Acacia koa*) [44], with ohia-lehua forming approximately 90% of the canopy [39]. Several species of minor mid-canopy woody plants also comprise the forest [44]. The 1900 and 1770 m sites were in degraded forest that was formerly used for cattle ranching, but had large trees that provided food and nesting sites, with introduced pasture grasses comprising most of the understory [44]. The 1650 m site had a more native understory, largely of ferns, which was generally rarely by native birds [39]. The study sites were approximately 1 km apart and recaptures of birds between sites were extremely rare, representing only 0.07% of 13,492 captures and recaptures. Because these sites were at upper elevations, mean monthly air temperature was cool [45], with occasional nighttime frost during the months October–March.

Eight native species of passerine birds, including six Hawaiian honeycreepers (Drepanidinae), along with the introduced Japanese white-eye, breed and molt in all study sites. Three endangered Hawaiian honeycreepers - the akiapolaau (*Hemiganthus monroi*), akepa, and Hawaii creeper (*Oreomystis mana*) – are specialized insectivores, but each uses a variety of foraging substrates [39]. The akiapolaau excavates wood and extracts beetle larvae. The akepa with its crossed bill can pry open ohia-lehua leaf buds and extract the microlepidoptera larva therein. The straight-billed Hawaii creeper probes for psocids, caterpillars, and spiders in crevices on the bark of trunks and branches of ohia-lehua. These and all other species of native birds glean arthropods from ohia-lehua foliage or twigs, and most consume nectar from ohia-lehua flowers. The Hawaii amakihi (*Hemignathus virens virens*), with its slightly decurved bill, is the most generalized forager in the community. The iiwi (*Vestiaria coccinea*) and apapane (*Himatione sanguinea*) have the reputation of being nectarivores, but stomach samples indicate that they also consume many insects and spiders [46], since the birds require protein for breeding and molting and ohia-lehua does not bloom intensively every month of the year. The two native non-honeycreepers are the Hawaii elepaio (*Chasiempis sandwichensis ridgwayi*), a monarchine flycatcher, and the omao (*Myadestes obscurus*), a thrush. The elepaio also gleans arthropods and the omao consumes fruit and gleans insects. These native species have clutch sizes of 2 eggs, based on size of family groups, and high annual adult survival ranging from 0.47 for the apapane to 0.85 for the akiapolaau [47].

The white-eye overlaps multiple foraging substrates with each native species, and spends much time gleaning arthropods on the same ubiquitous ohia-lehua foliage used by most native species [39]. The white-eye is an extreme generalist that consumes arthropods, nectar, and fruit [48], and is considered an exploitative competitor with several species of Hawaiian birds [43]. Its taxonomic family Zosteropidae is renowned for range expansion and niche diversification [49].

Arthropod prey from ohia-lehua foliage and twigs has biomass dominated by caterpillars and spiders [41], [50]. Highly relevant to timing and duration of molt is the seasonality of arthropod abundance. Most species of native birds actually breed during a seasonal decline of food, which begins in January and extends to July [41]. Food then increases to the seasonal high by September, when flowering in ohia-lehua generally peaks, and is maintained until January when the decline begins again [41]. At upper elevations, the birds initiate breeding during the cold winter and spring months (January–March) because food is relatively plentiful then, with an increasing photoperiod for foraging. They initiate molt during the seasonal low in food in June, but with compensating warm air temperatures [45]. Food gradually increases again during the molting period. The last two months of normal molt, September and October [41], occurred when arthropod prey was most abundant.

### Field methods

Birds were captured and banded in aerial and pole-based mist-nets during 1989–2006 at the 1900 m site and during 2004–2006 in the 1770 and 1650 m sites. An average of 2141 mist-net hours was operated per year, ranging from a low of 204 hours in 1998 to a high of 5323 hours in 2004. Molt was inspected and recorded for 6138 birds using standard ornithological techniques of spreading the wing and tail, moving a finger against the orientation of body feathers to feel and observe pin feathers, and blowing air against the orientation to observe pin feathers as described in [22]. Molt was recorded only by region (body, wing, and tail, where wing refers to remiges and tail to retrices) until 2002. Then, based on increasing detection of broken feathers [22], the identity of primary flight feathers being molted on each wing was identified and recorded. Age of bird was established by plumage or length of wing feathers, which are shorter in hatch year birds and second year birds before their first prebasic molt [42]. Breeding females were sexed by appearance of a brood patch. Active brood patches were identified by wrinkled bare skin with reddish tinge. Old brood patches were smooth with a grayish tinge and sometimes small pin feathers. Both sexes of the omao incubate, so females were identified by brood patch without a cloacal protuberance. Captures of akiapolaau were so rare that they were not used in all analyses.

### Statistical methods

Data from the 1900 m site were used for all analyses between time periods. We define normal molting by the months of high prevalence of molting adult birds. The initial analyses for time periods 1989–1999 and 2000–2006, conducted separately, used individual birds once on their initial capture, with species, year, and month as factors with all interactions in a logistic regression. Year was also modeled as trend during each time period. Then we define the months of extended and early molt by patterns detected in individual birds recaptured during the months of low prevalence of molting. Focused analyses of changes of prevalence of extended and early molt for different age groups were performed using species, time period, and the interaction in logistic regression. Time period was added last to the model as the factor of main interest. A bird was only used once within a time period and was considered extended or early molting if it molted as such at least one year in the time period. This procedure preserves the independence of samples and the estimated prevalence of early molt or extended molt is based on the number molting versus the number that could have been molting. We tested for trend of extended molt and early molt, and the correlation between them, using annual prevalence of each. Here an individual bird was used at most once per year. Analysis of deviance tables are provided for all logistic regressions, with effect, degrees of freedom (Df), deviance, residual Df, residual deviance, and p-value for the Chi-square test of the significance of the effect. All correlation analyses used the Pearson test.

We compared molting data in all three study sites during 2005–2006, when banding was conducted in the same months at all sites. Aerial mist-nets were still being erected in the 1650 m and 1770 m sites during 2004, so banding was not consistent that year. The logistic regression included year, species, and site, in that order, with all interactions.

We also investigated suspended molt of birds during the months of July, August, and September, the months of main molting. Suspended molt was evident in birds that were molting body feathers only during these months, and was proved by recaptures of birds. Prevalence was estimated in relation to birds that were molting primary flight feathers. Individual birds were used just once for this analysis. Prevalence of suspended molt was tested with logistic regression using species, month, and time period as effects with all interactions.

Birds that were captured more than once were analyzed separately. A bird could have consistent normal molt, consistent extended or early molt, or variable molt. We used contingency table analysis to determine if these proportions differed for birds between the two time periods.

We compared breeding and molting of female birds during June, the last month of the breeding season, between the two time periods. Females that month were classified as molting with an active brood patch, molting with an old brood patch, not molting with an active brood patch, and not molting with an old brood patch. We used multinomial regression using class of molt as the dependent variable with species and time period as independent variables.

It is possible that changes in molt could be affected by change in breeding seasonality. Since June is the last month when active nests were discovered, we compared the prevalence of females with active brood patches during June and July for the two time periods. We also compared the prevalence during the earliest month in which females with active brood patches were detected for each species. We then compared the prevalence of molt-breeding overlap both at the beginning and end of the breeding season using females with active brood patches that were molting.

Our documentation of timing of particular primary feather molt was restricted to 2002 and later. With all the changes in molt that we document, we could only use modal patterns of primary molt each month and only three species (akepa, amakihi, iiwi) had sufficient sample size each month during June to October. Limited data for some months were available for the apapane and creeper.

We determined how changes in molt contributed to survival by comparing survival of adult birds with extended molt during January–March in 2000 to 2004 and those without molt during those months. We defined survival as those individuals that were recaptured at least one year later. Only birds from 2000 to 2004 could be used to ensure sufficient opportunity for recapture at least one year later because our research was terminated in 2006 by the U.S. Fish and Wildlife Service. Only five native species were captured during each month each year. Individual birds were used once per year for the best estimate of prevalence of extended molt for a given year. We used a logistic regression of survival in a model with species, year as trend, and molting status, with all interactions. As a control for this method potentially reducing survival for birds captured later in 2000–2004, with less opportunity to be recaptured, we performed a similar analysis for the same five species of birds captured during 1990–1994 with possible recapture in 1995–1996. Just as there were fewer mist-net hours operated in 2005–2006 (4099.4) than in 2004 (5322.9), there were fewer hours operated in 1995–1996 (446.7) than in 1994 (653.4). No trend during 1990–1994 would imply a trend detected during 2000–2004 was real.

Analysis of recapture of birds with asymmetric molt was based on three species which each had sufficient samples of survivors and non-survivors. A score was provided for each individual that expressed the difference of the number of the asymmetric feather of one wing from the closest primary number being molted in the other wing, summed over the number of asymmetric feathers. An analysis of variance of molt score was used to test the effect of species and survival.

To determine the overall molt changes for individual species and months, we calculated the difference in prevalence of molt between time periods for each month for each species. A preliminary analysis of variance of the differences revealed significant month and species effects. T-tests were then used to determine which months and species had mean differences greater than 0.

We also analyzed mean monthly air temperatures, because a change in temperature could produce food limitation by affecting the production of arthropod prey in ohia-lehua and koa trees. Data were collected from a weather station located at 1940 m in the center of the refuge. Data were available as mean daily air temperatures from the refuge during 1993 and first half of 2003 and were downloaded (mesowest.utah.edu) for the rest of 2003 through 2006. Monthly means for each year were calculated from temperatures logged every 15 minutes. Mean monthly air temperatures were then analyzed using analysis of variance with month and year as factors, along with the interaction.

## Results

### Normal molt before 2000

**Figure 1:**
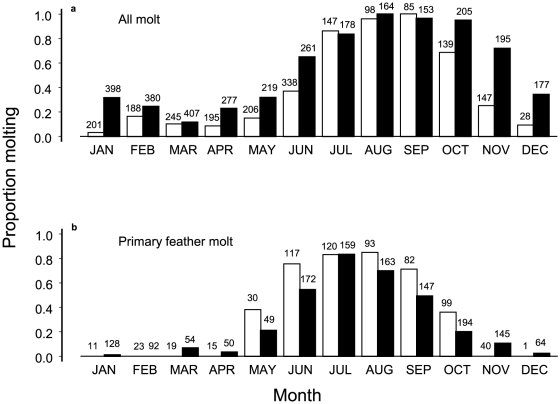
Overall changes in monthly molt between time periods. (a) Proportion of birds of all native species during initial capture molting each month during 1989–1999 (white bars) and 2000–2006 (black bars). (b) Proportion of molting birds that were molting primary flight feathers. Sample sizes are above bars. Analysis of deviance from logistic regression for any molt is in Table 1 and for primary molt is in Table 2.

**Figure 2:**
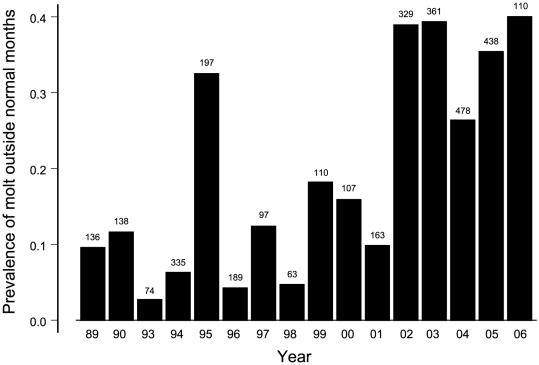
Proportion of birds of all native species molting during non-normal months of November through May by year. Gaps represent years with insufficient data. Individual birds were used only once per year. Sample sizes are above bars. This reveals the significant year effect in Table 1. There is no trend during 1989–1999, but significant trend during 2000–2006.

For years before 2000 in the 1900 m study site, the main molting period generally began in June and ended in October for all species (Fig. 1a, Table 1). The months with high prevalence of molt included molt of primary flight feathers (Fig. 1b, Table 2). None of 93 birds, molting on their initial capture during November–April, were molting primary feathers. Only 3 of 92 recaptured birds were molting primaries during these months (one iiwi, akiapolaau, and omao). Birds nesting in June do not usually molt as reflected by the lower prevalence of molting of birds in June than July. Most individuals finished molting during October. Some birds were molting outside of these normal months, with prevalence that varied with year (Fig. 2, Table 1), but without trend over the time period when year was modeled as a covariate (Fig. 2, trend, p = 0.41). There was no correlation between annual prevalence of molt during November–May and mist-net hours which ranged from 204 to 2600 (r = −0.05, t_7_ = 0.14, p = 0.90).

**Table 1:** Analysis of deviance for molt for different time periods.

Effect	Df	Deviance	Residual Df	Residual Deviance	P-value
Years 1989–1999					
Null			1490	1864.59	
Species (S)	7	37.08	1483	1827.51	<0.0001
Month (M)	11	663.44	1472	1164.07	<0.0001
Year (Y)	8	124.79	1464	1039.29	<0.0001
S×M	70	127.56	1394	911.73	<0.0001
S×Y	45	38.56	1349	873.17	0.74
M×Y	57	104.78	1292	768.40	0.0001
S×M×Y	129	56.73	1163	711.67	1.00
Years 2000–2006					
Null			1772	2450.69	
Species (S)	7	37.43	1765	2413.26	<0.0001
Month (M)	11	790.57	1754	1622.69	<0.0001
Year (Y)	6	28.69	1748	1594.00	<0.0001
S×M	68	102.27	1680	1491.72	0.005
S×Y	35	51.81	1645	1439.91	0.03
M×Y	60	154.38	1585	1285.53	<0.0001
S×M×Y	165	124.03	1420	1161.50	0.99

**Table 2:** Analysis of deviance of molt of primary flight feathers.

Effect	Df	Deviance	Residual Df	Residual Deviance	P-value
Null			1434	1925.53	
Species	7	55.59	1427	1879.94	<0.0001
Month	11	630.26	1416	1249.67	<0.0001
Time period	1	7.59	1415	1242.08	0.006
Species×month	68	75.11	1347	1166.97	0.26
Species×period	7	22.22	1340	1144.74	0.002
Month×period	11	21.25	1329	1123.49	0.03
Species×month×period	44	20.07	1285	1103.42	0.99

Repeated captures of 130 birds outside of the normal molting months during both time periods defined extended and early molting. Extended molting occurred when a bird is still molting during the months November–March and not later in the series or during April or May. There were 75 cases representing all native species that fit extended molt, and 24 cases where the bird was recaptured later in November–March, after extended molt had been documented, but with fewer body regions in molt, indicating that molt was finishing. Early molt occurred when a bird was not molting during months November–March, but molted during months April or May. There were 21 cases in most native species that fit early molt, including 8 cases where early molt started earlier in February or March. These were mostly during 1995, the most unusual year during 1989–1999 (Fig. 2).

**Figure 3:**
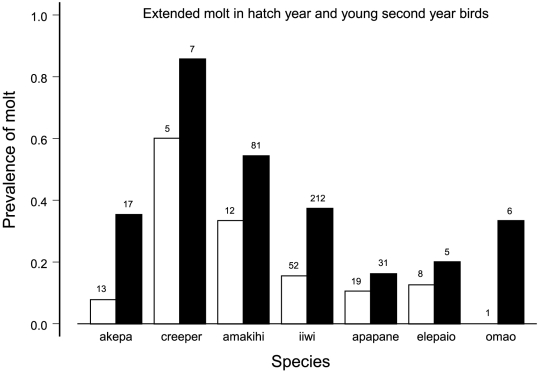
Change in molting patterns by combined hatch year birds during November and December and second year birds during January–March. Individual birds were used only once. Open bars represent years 1989–1999, black bars 2000–2006. Sample sizes are above bars. Analysis of deviance from logistic regression is in Table 3.

Most hatch year birds finished their juvenile molt by October, but some extended their molt into November and December, even as late as March of their second year, with significant variation among species (Fig. 3, Table 3). For example, only 3 species (iiwi, akepa, elepaio) had second year birds molting in February. Two thirds of these cases occurred in 1995, again an unusual year as discussed later. All the rest except one in 1989 occurred in 1997.

**Table 3:** Analysis of deviance table for logistic regression of changes in molt of hatch year birds during November and December and as second year birds during January through March of the following year.

Effect	Df	Deviance	Residual Df	Residual Deviance	P-value
NULL			310	414.78	
Species	7	28.06	303	386.73	0.0002
Time period	1	15.34	302	371.39	0.0001
Species×time period	6	3.61	296	367.79	0.73

**Figure 4:**
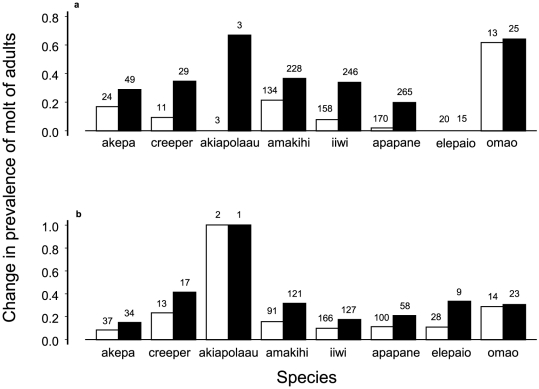
Change in molting patterns by adult birds. Nov–Mar are months for extended molt (a), Apr–May for early molt (b). White bars are for 1989–1999, black bars for 2000–2006. Sample sizes are above bars. Analyses of deviance from logistic regression are in Table 4. Individual birds were used only once for each analysis.

The pre-basic molt of adults was similarly extended from October to March with significant variation among species (Fig. 4a, Table 4). There was evidence from banding records of individual birds showing extended molting before 2000. A creeper was molting in June 1989 and still molting in March 1990, while an amakihi was molting in June 1996 and still molting in November 1996. In addition, all species of native birds had adults that began their pre-basic molt early during April or May, with varying prevalence (Fig. 4b, Table 4). Table 5, for birds with repeated captures over years, shows that before 2000 extended molt was never and early molt was rarely repeated.

**Table 4:** Analyses of deviance for logistic regression of extended and early molt of adults.

Effect	Df	Deviance	Residual Df	Residual Deviance	P value
Extended molt					
Null			1390	1490.71	
Species (S)	7	88.75	1383	1401.96	<0.0001
Time period (TP)	1	76.75	1382	1325.21	<0.0001
S×TP	7	19.60	1375	1305.61	0.007
Early molt					
Null			840	791.73	
Species (S)	7	32.30	833	759.43	<0.0001
Time period (TP)	1	16.46	832	742.97	<0.0001
S×TP	7	1.59	825	741.37	0.98

**Table 5:** Proportions of extended, early, and normal molt of individual birds captured during more than one year.

Type of molt	Time period	Pattern of recaptures		
		Repeated type	Repeated normal	Mixed molt
Extended	1989–1999 (n = 50)	0.00	0.88	0.12
Extended	2000–2006 (n = 201)	0.07	0.40	0.53
Early	1989–1999 (n = 16)	0.06	0.88	0.06
Early	2000–2006 (n = 57)	0.07	0.74	0.19

Molt-breeding overlap was not common in June before 2000 (Table 6). Most birds with active brood patches were not molting. Molt-breeding overlap was limited to body feathers except for one creeper in 1989. Molt-breeding overlap was almost non-existent at the beginning of the breeding season in all but two species (iiwi and creeper), together having less than 4% prevalence. The only strong deviation from this pattern occurred in 1995 when 7 of 8 native species initiated molt during March–May, including females with active brood patches.

**Table 6:** Breeding and molting status at the end of the breeding season^1^.

Species	Active BP, No molt	Old BP, No molt	Old BP, Molt	Active BP, Molt
Akepa (18/11)	0.56/0.36	0.22/0.27	0.06/0.27	0.17/0.09
Creeper (6/6)	0.83/0.17	0.00/0.00	0.17/0.50	0.00/0.33
Amakihi (33/34)	0.55/0.35	0.12/0.06	0.27/0.06	0.06/0.29
Iiwi (44/21)	0.64/0.38	0.09/0.00	0.18/0.38	0.09/0.24
Apapane (21/16)	0.67/0.81	0.05/0.13	0.10/0.00	0.19/0.06
Elepaio (6/2)	0.83/1.00	0.17/0.00	0.10/0.00	0.00/0.00
Omao (2/7)	1.00/0.71	0.00/0.00	0.00/0.00	0.00/0.29

1 BP represents brood patch. Values are proportions of individuals in each status during 1989–1999 before the slash, during 2000–2006 after the slash. Sample sizes for each time period follow species names.

Assuming that the pattern of primary molt did not change during 2002–2005, all species followed the same pattern of first molting inner primaries with sequential molting to the outermost primary (Table 7). The longer outer primary feathers 6–9 were molted over several months compared with shorter feathers 1–5 that were generally molted in two sets, each requiring a single month. The akepa and amakihi appear to molt the longer outer primaries more slowly than the larger iiwi and apapane.

**Table 7:** Identity of the most common primary feathers molted each month. The dash indicates insufficient sample size to identify the mode.

	Month				
Species	June	July	August	September	October
Akepa	1–3	4–6	7–9	8–9	9
Amakihi	1–5	6–8	6–8	8–9	8–9
Iiwi	1–3	4–6	5–6	7–9	7–9
Creeper	1–2	-	9	-	-
Apapane	-	-	6	7	-

Suspended molt was detected before 2000 in all species. This was indicated by lower proportions of individuals molting primary feathers than those molting any feathers during the months of July–September (Fig. 1a,b). Suspended molt occurred in 23% of the 226 individual birds inspected during these months (data shown below with other time period).

### Changes in timing, duration, and symmetry of molt during 2000–2006

**Figure 5:**
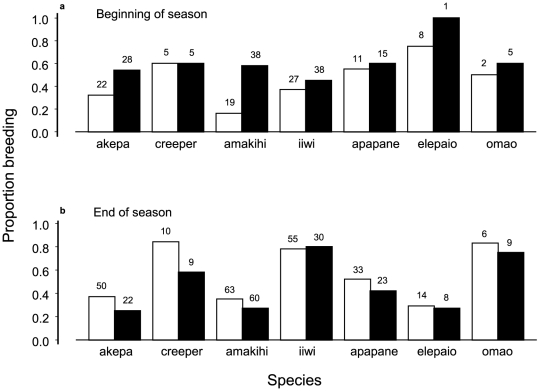
Proportion of birds breeding early and late in the season. Earliest breeding months are in (a); last two breeding months are in (b). White bars indicate proportions during 1989–1999; black bars during 2000–2006. Sample sizes are above bars. Analyses of deviance from logistic regression are in Table 8.

There were only minor changes in the timing and duration of the breeding season between the two time periods. Only one species in 2000–2006 initiated nesting earlier than during 1989–1999 (one month earlier by one individual), and no species ended the season later. However, beginning in 2000 and extending to 2006, a higher proportion of females initiated breeding during the first normal month of the season and a lower proportion were breeding at the normal end of the season (Fig. 5, Table 8). There was simply a shift within the former breeding season.

**Table 8:** Analysis of deviance table for breeding early and late in the season.

Effect	Df	Deviance	Residual Df	Residual. Deviance	P-value
Breeding early					
Null			13	20.17	
Species (S)	6	6.85	7	13.13	0.33
Time period (TP)	1	7.98	6	5.33	0.005
S×TP	6	5.33	0	0.00	0.50
Breading late					
Null			27	116.33	
Species (S)	6	51.18	21	65.14	<0.0001
Month (M)	1	41.94	20	23.20	<0.0001
Time period (TP)	1	5.53	19	17.67	0.019
S×M	6	5.30	13	12.37	0.505
S×TP	6	2.19	7	10.18	0.901
M×TP	1	0.98	6	9.20	0.323

In contrast, the timing and duration of molt changed drastically beginning in 2000. Using the same logistic regression on molt in birds upon initial capture during 1989–1999, all factors retained their significance with an additional species by year interaction (Fig. 1a, Table 1). However, during 2000–2006 there were six months when 50% or more of birds were molting, compared with only four such months during 1989–1999 (Fig. 1a). The additional months were June and November, at each end of the set of previous normal months. Moreover, there was a significant trend of birds molting during the non-normal months between 2000 and 2006 (Fig. 2, trend, p = 0.02), whereas no trend existed before 2000. There was no correlation between annual prevalence and mist-net hours which ranged from 721 to 5323 (r = 0.42, t_5_ = 1.24, p = 0.35). In fact, 2006 had the highest prevalence of any year even though it only included the months of January–May with the lowest mist-net hours (Fig. 2).

The changes in molt affected individual birds in every age class. More hatch-year and young second-year birds of every species took longer to complete molt (Fig. 3, Table 3). Adults took longer as well (Fig. 4, Table 4). Adults of only 3 species had extended molt in February before 2000 but 7 species had such molt after 1999. Some direct evidence of the change came from 9 adults in 5 species (akepa, creeper, amakihi, iiwi, and omao) during 2000–2005. These were captured in June beginning molt and later recaptured during the months November–March while still molting.

Body feathers were mainly molted during the extended period, although the molting of flight feathers was also extended in adults (Fig. 1b). The number of months during which primary feathers were molted more than doubled between time periods (Fig. 1b). In particular, only one iiwi molted primary feathers during November before 2000 (n = 23, prevalence = 0.043), but five species (akepa, creeper, amakihi, iiwi, and apapane) did so after 1999 with prevalence ranging from 0.10 to 1.00 (n = 105, binomial test based on 0.043, p<0.0001). There was no change in prevalence of primary feather molt in June for these species, with pairs defined by species for proportion molting before 2000 and after 1999 (paired t_5_ = 2.03, p = 0.10).

Adult birds also initiated molt earlier beginning in 2000 (Fig. 4b), as indicated by higher prevalence of molt during April and May (Fig. 4b, Table 4). Adults of 6 native species had higher prevalence of both extended and early molt, with variability among species (Fig. 4, paired t = 0.08, df = 7, p = 0.94). Half of the 8 species more than doubled their prevalence of extended molt while only 2 doubled prevalence of early molt over the previous time period. Three adult akepa and two amakihi in 2004–2005 were early molting in April or May and still molting in November (2 cases), December (1 case), and January (2 cases).

Young birds had extended molt followed by early molt, and included 2 iiwi, 3 amakihi, and 1 akepa during 2003–2005. These individuals started their pre-basic molt within one or two months of completing their juvenile molt.

Extended molt was not consistent for individual birds among years. During 2000–2006, there were cases of repeated extended molt and normal molt, but mostly cases of switching between extended and normal molt. Contingency table analysis indicated different patterns of extended molt between time periods (Table 5, Χ^2^ = 37.45, df = 2, p<0.0001). Early molting occurred more after 1999 (Fig. 4b), but proportions from repeated recaptures remained the same (Table 5, Χ^2^ = 1.62, df = 2, p = 0.45).

**Figure 6:**
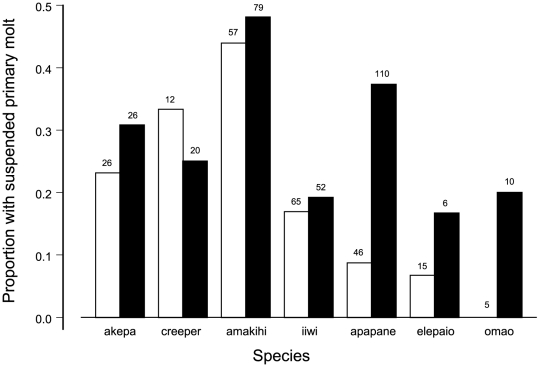
Suspended molt of primary flight feathers during the peak molting months of July–September. Years 1989–1999 represented by white bars, 2000–2005 by black bars. Individual birds were used only once. Values are proportion of birds molting body feathers that were not molting flight feathers. Sample sizes are above bars. Analysis of deviance from logistic regression is in Table 9.

Suspended molt of primary flight feathers by birds that were molting small body feathers during the main molting months of July–September increased in prevalence in 6 of 7 species after 1999 (Fig. 6, Table 9). Several records of birds illustrate suspended molt (Table 10). Based on the particular primaries molted at that time it would have been impossible for them to have completed molt by the second capture, or to have started molt after the first capture (Table 10), based on the sequence of molt of primary flight feathers (Table 7).

**Table 9:** Analysis of deviance of suspended primary molt during July–September.

Effect	DF	Deviance	Residual Df	Residual Deviance	P- value
NULL			37	133.40	
Species	6	5.97	31	127.44	0.43
Month	1	0.49	30	126.51	0.48
Time period	1	5.38	29	121.57	0.02
Species×month	6	15.61	23	105.95	0.02
Species×period	6	12.75	17	93.20	0.047
Month×period	1	3.00	16	90.20	0.08
Species×month×period	5	9.59	11	80.61	0.08

**Table 10:** Evidence of suspended molt from recaptured birds.

Species	First capture	Molt first capture	Recapture	Days elapsed	Molt recapture
Iiwi	7/9/2005	Primaries 2–4	8/30/2005	52	No primary molt
Iiwi	7/23/2005	No primary molt	8/9/2005	17	Primary 6
Apapane	8/17/2004	No primary molt	9/21/2004	34	Primaries 7–8
Apapane	8/25/2004	No primary molt	9/2/2004	8	Primary 8
Amakihi	8/17/2004	Primary 6	9/18/2004	31	No primary molt

Prevalence of both extended molt and early molt increased during 2000 to 2006 and were positively correlated (r = 0.78, t_5_ = 2.82, p = 0.04). Year 2004 was lower than the two preceding and the two following years (Fig. 2; t_3_ = 11.68, p = 0.002), an important result for identifying the cause of the changes in molt. The change in molt seasonality and lack of change in breeding seasonality induced molt-breeding overlap both early and late in the breeding season, with correlation among species (r = 0.84, t_5_ = 3.52, p = 0.02). The interaction model in multinomial regression was supported for molt/no molt and active/old brood patches at the end of the season (Table 6, likelihood ratio test = 31.5, p = 0.025). Four species had an increase in molt-breeding overlap (amakihi, creeper, iiwi, omao, with the greatest change in creeper from 0 to 0.40 of females). Three species had a slight decrease or no change (akepa, apapane, elepaio).

**Figure 7:**
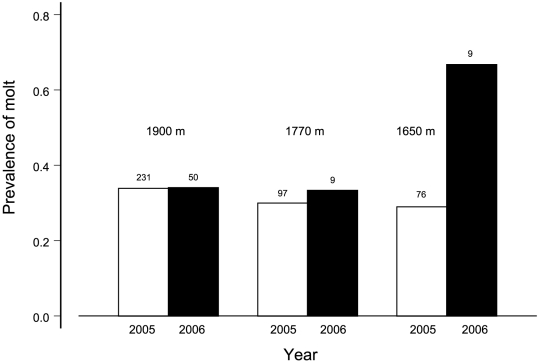
Combined extended and early molting during 2005–2006 at three study sites. Analysis of deviance from logistic regression is in Table 11. Sample sizes are above bars.

Molting during the main months, July–October, was similar among the three sites (test of proportions, Χ^2^ = 3.99, df = 2, p = 0.14). The site comparison for the non-normal months of November–May had a significant three-way interaction between site, species, and year (Table 11). The site by year component is based on the 1650 m site having lowest prevalence in 2005 and highest prevalence in 2006 (Fig. 7). Only four species were captured during both years (creeper, iiwi, apapane, amakihi). These contributed to the interaction in that 4 of 4, 1 of 4, and 3 of 4 increased between years at the 1650, 1770, and 1990 m sites, respectively.

**Table 11:** Comparison of extended and early molt among sites.

Effect	Df	Deviance	Residual Df	Residual Deviance	P- value
NULL			476	601.54	
Species	7	23.95	469	577.58	0.001
Year	1	0.73	468	576.86	0.39
Site	2	1.03	466	575.82	0.60
Species×year	6	3.48	460	572.35	0.75
Species×site	12	10.80	448	561.55	0.55
Year×site	2	2.54	446	559.00	0.28
Species×year×site	8	15.99	438	543.01	0.04

**Figure 8:**
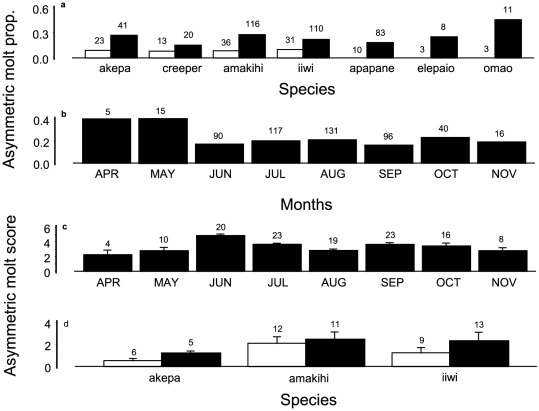
Asymmetric molting of primary flight feathers during 2002–2005. Sample sizes above bars in all panels: (a) by species, year 2002 in white bars, years 2003–2005 in black bars, showing increase over time; (b) by month. Asymmetric molting scores by month (c) and survival (d). Open bars indicate survivors, black bars non-survivors. Sample sizes above bars. Analysis of deviance for asymmetric molting prevalence from logistic regression is shown in Table 12. Analysis of variance table for molt score is shown in Table 13 for year and month, and in Table 14 for survival.

Asymmetric molt was first detected in August 2002. There was no molt of primary feathers during January–May of 2002 in 217 birds inspected and no asymmetric molt was evident in 48 birds molting primary feathers in June or July of 2002. Primary molt before June began in 2003. The evidence thus points to asymmetric molt first starting in August 2002. All species had increased prevalence of such molt after 2002, with no difference among months, which was sustained through 2005 as the last year when birds were inspected each month (Fig. 8, Table 12). There was no difference in asymmetric molt scores among species or years, but June, when most birds started molting, had the highest scores (Fig. 8, Table 13). For individuals with asymmetric molt, there was no difference in molt score between survivors and non-survivors (Fig. 8, Table 14). However, high scores of 6 to 8 were concentrated in non-survivors. Some non-surviving individuals were even repeating molt of inner primaries while they were still molting outer primaries.

**Table 12:** Analysis of deviance table of prevalence of asymmetric molt.

Effect	Df	Deviance	Residual Df	Residual Deviance	P-value
Null			123	163.62	
Species	7	5.86	116	157.76	0.56
Month	7	5.18	109	152.58	0.64
Year	3	44.40	106	108.18	<0.0001
Species×year	18	24.25	88	83.93	0.15

**Table 13:** Analysis of variance table for asymmetric molt score during 2002–2005.

Effect	Df	Sum of squares	Mean square	F statistic	P-value
Species	7	10.27	1.47	0.95	0.47
Year	3	3.61	1.20	0.78	0.51
Month	7	59.74	8.53	5.54	<0.0001
Residual	105	161.90	1.54		

**Table 14:** Analysis of variance of asymmetric molt score in relation to species and survival.

Effect	Df	Sum of squares	Mean square	F statistic	P-value
Species	2	15.55	7.78	1.76	0.18
Survive	1	6.98	6.98	1.59	0.21
Species×survive	2	1.41	0.70	0.16	0.85
Residual	50	220.27	4.41		

### Survival consequences of changes in molt

**Figure 9:**
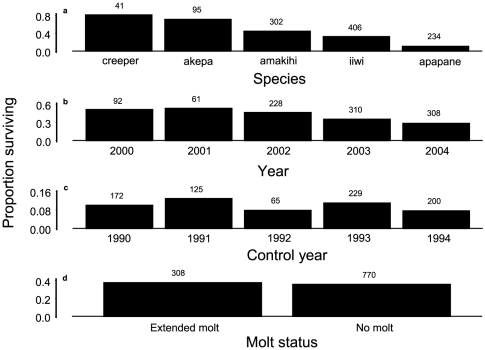
Survival of adult birds captured during January–March. Panels a, b, and d are for years 2000–2004. Panel c is for control years 1990–1994. Sample sizes are above bars. Logistic regression modeled survival based on species, year as trend, and molting status (extended molt or no molt), with just species and year modeled as trend for the control years. Analyses of deviance from logistic regression are in Table 15.

Logistic regression showed that adult survival of birds captured during January to March declined between 2000 and 2004 but not with molting status as a main effect (Fig. 9, Table 15). The control years showed that the declines were not based on captures later in the time frame (Table 15). The interactions involved the akepa as the only species with higher survival of individuals with extended molt. The creeper and apapane with extended molt had lower survival.

**Table 15:** Analysis of deviance of survival of birds captured during January through March in 2000 to 2004, and in control years 1990 to 1994.

Effect	Df	Deviance	Residual Df	Residual Deviance	P-value
Years 2000–2004					
NULL			49	251.91	
Species (S)	4	171.78	45	80.13	<0.0001
Year (Y)	1	15.92	44	64.21	<0.0001
Molt status (M)	1	0.04	43	64.17	0.84
S×Y	4	11.24	39	52.93	0.02
S×M	4	7.89	35	45.04	0.10
Y×M	1	1.90	34	43.14	0.17
S×Y×M	4	11.10	30	32.04	0.03
Years 1990–1994					
NULL			23	53.97	
Species	4	20.06	19	33.91	0.0005
Year	1	1.43	18	32.49	0.23
Species×year	4	5.50	14	26.99	0.24

### Overall changes in molt by species and month

**Figure 10:**
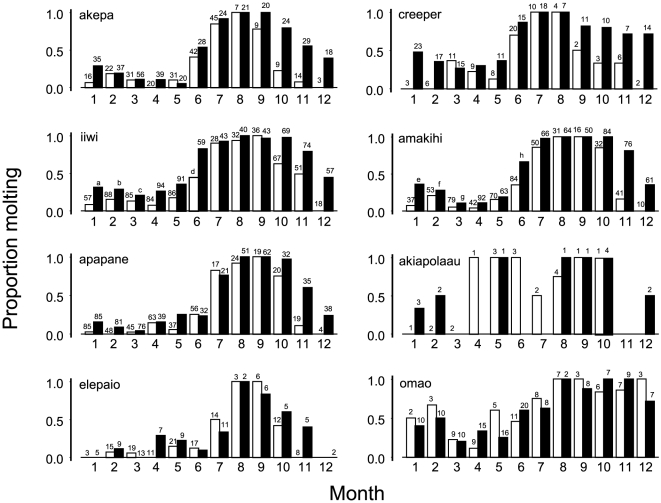
Summary of molt each month for each species during the two time periods. White bars are for 1989–1999, black bars for 2000–2006. Sample sizes above bars in all panels. The letters a–h represent sample sizes too large for the width of the bars: a = 193, b = 163, c = 155, d = 132, e = 154, f = 126, g = 117, h = 108.

Figure 10 shows the differences in molt of each native species for each month between time periods. The mean differences in molt were significantly greater than 0 for the 6 honeycreeper species but not for the elepaio or omao. The creeper had the largest change in molt (0.26, p = 0.003) followed by the akiapolaau (0.23, p = 0.04), amakihi (0.20, p = 0.001), akepa and iiwi (0.18, p = 0.01), and apapane (0.17, p = 0.02) Six species molted in months that were previously not used. The significant monthly differences in molt between time periods were November (0.41, p = 0.01), December (0.33, P<0.001), October (0.26, p = 0.03), June (0.16, p = 0.01), and April (0.14, p = 0.04).

### Air temperature between time periods

**Figure 11:**
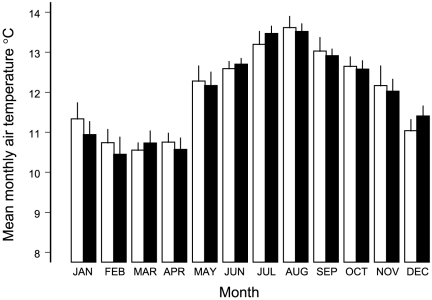
Mean monthly air temperatures between time periods. Years 1993–1999 are in white bars, 2000–2006 in black bars. Thin lines are standard errors. Analysis of variance is in Table 16.

Monthly air temperature varied seasonally, but without difference between time periods and with no interaction between month and time period (Fig. 11, Table 16).

**Table 16:** Analysis of variance of mean monthly air temperatures between 1993–1999 and 2000–2006.

Effect	Df	Sum of squares	Mean square	F value	P-value
Month	11	181.43	16.49	26.95	<0.0001
Time period	1	0.05	0.05	0.09	0.77
Month×time period	11	1.95	0.18	0.29	0.99
Residuals	142	86.91	0.61		

## Discussion

Hawaiian birds show the same progressive molt of primary flight feathers from inner to outer as other birds [51], but the changes in timing, duration, and symmetry of molt are unprecedented in wild populations. The change in prevalence of early and extended molting, without change in breeding seasonality, accounts for molt-breeding overlap both at the beginning and end of the breeding season. The change included more birds suspending molt of their primary flight feathers during the main molting months and extending this molt into months never or rarely used before. The changes in molt occurred in parallel with community-wide stunted growth of young birds, also unprecedented [40]. The changes in molt are more diverse and address major issues in life history of tropical birds, as well as conservation of Hawaiian birds.

### Molt in tropical birds

In most years before 2000, the molting pattern of Hawaiian birds was similar to that of other tropical birds where molt predictably starts at the same time each year following breeding [52]–[53]. Normal molt at Hakalau was also similar to molt of Hawaiian birds documented elsewhere in earlier studies [54]–[55], but with less molt-breeding overlap [55]. The variation in molt among species of Hawaiian birds in the same community is much less than that documented within much more taxonomically diverse neotropical or paleotropical communities [34], [37], [52]–[53], [56]–[57]. Even so, the patterns during both time periods address major problems in understanding molt in tropical birds.

Hawaiian birds and diverse tropical birds have tails of low molting prevalence at both ends of the monthly distribution of molt within the population [34], [52]–[53], [56]–[57], [58]. One interpretation is that the tails represent out of phase molt within the population stemming from a long breeding season. Our study shows that these tails are small amounts of early molting and extended molting. The early molt in 1995 was associated with lowest food level between 1994 and 1997 [41], when an irruption of introduced yellow-jacket wasps (*Vespula pensylvanica*) occurred before they were subsequently controlled. These tails expanded again, but in both directions, after 2000 with chronic food limitation throughout the year [22], [39]–[40]. The tails in the distribution of molt in other tropical birds, less drastic than these, might then indicate food limitation during the molting season. Such limitation is assumed by the seasonality hypothesis for low reproductive rate, with adult populations regulated by food during the non-breeding season [59]–[60]. In this context, the white-eye/Hawaiian bird situation may provide an example of why tropical birds do not molt when migratory birds are present [61].

The timing of molt of particular primary flight feathers may contribute to the length of the molting season [62]. Outer primary feathers are larger than the inner primaries and thus more expensive to replace. In addition, loss of these feathers has consequences for flight. Less thrust can be generated and rapid evasive maneuvering may be more difficult [23]. These problems may have survival consequences in environments where more predators are present in different planes of the forest (Hawaiian birds have only a single rare native predator, the Hawaiian hawk [*Buteo sandwichensis*]). If replacement of outer primaries is related to predation, then comparison of locations that vary in predation pressure should generate a correlation between time to replace outer primaries and the risk of predation.

The least understood problem in molt of tropical birds is the lack of intra-population synchrony within the main molting months. This has been documented throughout the world [29], [34], [52]–[53], [56]–[58], with percentages ranging from around 50% [52] to 80% [53]. This was also initially explained by individuals in the population being out of phase from the long breeding season [52]–[53]. Compared with other tropical birds, Hawaiian birds have the highest percentage of birds molting at a time when all feathers are considered (95% versus 80% [53]), but match some tropical birds at 80% when attention is restricted to primary flight feathers. We have shown that the phenomenon of birds not molting represents suspended molt, which increased in prevalence for all species. It is recognized that molt can be suspended and needs to be identified by the length and wear of flight feathers [63]. Suspended molt may be the major determinant of the length of molting seasons of tropical birds.

### Implications for conservation

The extensive changes in molt are associated with chronic food stress. Four lines of evidence support the hypothesis that competition for food from increased numbers of white-eyes caused the stress. First, the collective differences in molt between 1989–1999 and 2000–2006, coincident with the stunting of growth of native species, occurred with increased numbers of white-eyes [39]–[40]. Second, the difference in molt between birds in the open forest study sites and the 1650 m site during 2005 was associated with a five-fold higher capture rate of white-eyes in the two upper elevation sites [40]. The 1650 m site had birds with more normal growth [40] and the lowest non-normal molt during 2005. Third, the change in prevalence of extended molt in the 1650 m site to the highest among sites in 2006 coincided with the continuing increase of white-eye density in that area of the refuge (2.67 to 2.83 birds/ha) and lowered density in the area with the other study sites (4.42 to 3.65) [64]. Fourth, there is the anomalously lowest value in non-normal molt for year 2004 relative to 2002–2003 and 2005–2006 in the 1900 m site (Fig. 2). The anomaly disappears under the competition hypothesis because year 2004 had the only negative residual of white-eye captures per net hour during 2000–2006 [39].

We have shown elsewhere that yellow-jacket wasps, parasitoid wasps, chewing lice, mammalian predators, and avian malaria could not account for the contrasts in space and time of food limitation on native bird species [22], [39]–[40], [42]. Here we showed that air temperature was unlikely to have lowered productivity of arthropods in tree canopies.

Because early and extended molt increased during 2005 and 2006, the environment worsened after 2004. The lower survival of species during the cool months of 2000–2004 suggests that food was so limiting that it overwhelmed any adaptive aspect of extended molt. The lower survival matches the community wide declines observed in our study sites by 2006 [39], and to refuge-wide declines based on survey data between 2000 and 2007 [47]. The molting problems of the birds are the clearest signal that management of food, likely through selective control of white-eyes, is essential for native bird recovery. Outcomes of successful management that ameliorates food limitation would be less early molt, less extended molt, less suspended molt, elimination of asymmetric molt, normal growth of young birds, more fat in adults and young, and elimination of major fault bars.
